# Community-based medication delivery program for antihypertensive medications improves adherence and reduces blood pressure

**DOI:** 10.1371/journal.pone.0273655

**Published:** 2022-09-09

**Authors:** Dan N. Tran, Kibet Kangogo, James A. Amisi, James Kamadi, Rakhi Karwa, Benson Kiragu, Jeremiah Laktabai, Imran N. Manji, Benson Njuguna, Daria Szkwarko, Kun Qian, Rajesh Vedanthan, Sonak D. Pastakia

**Affiliations:** 1 Department of Pharmacy Practice, Temple University School of Pharmacy, Philadelphia, Pennsylvania, United States of America; 2 The Academic Model Providing Access to Healthcare (AMPATH), Eldoret, Kenya; 3 Department of Family Medicine, Medical Education and Community Health, Moi University School of Medicine, Eldoret, Kenya; 4 Department of Pharmacy Practice, Purdue University School of Pharmacy, Indianapolis, Indiana, United States of America; 5 Department of Clinical Pharmacy and Practice, Moi Teaching and Referral Hospital, Eldoret, Kenya; 6 Department of Family Medicine, Warren Alpert Medical School of Brown University, Providence, Rhode Island, United States of America; 7 Department of Population Health, NYU Grossman School of Medicine, New York, United States of America; Harvard Medical School, UNITED STATES

## Abstract

Non-adherence to antihypertensive medications is a major cause of uncontrolled hypertension, leading to cardiovascular morbidity and mortality. Ensuring consistent medication possession is crucial in addressing non-adherence. Community-based medication delivery is a strategy that may improve medication possession, adherence, and blood pressure (BP) reduction. Our program in Kenya piloted a community medication delivery program, coupled with blood pressure monitoring and adherence evaluation. Between September 2019 and March 2020, patients who received hypertension care from our chronic disease management program also received community-based delivery of antihypertensive medications. We calculated number of days during which each patient had possession of medications and analyzed the relationship between successful medication delivery and self-reported medication adherence and BP. A total of 128 patient records (80.5% female) were reviewed. At baseline, mean systolic blood pressure (SBP) was 155.7 mmHg and mean self-reported adherence score was 2.7. Sixty-eight (53.1%) patients received at least 1 successful medication delivery. Our pharmacy dispensing records demonstrated that medication possession was greater among patients receiving medication deliveries. Change in self-reported medication adherence from baseline worsened in patients who did not receive any medication delivery (+0.5), but improved in patients receiving 1 delivery (-0.3) and 2 or more deliveries (-0.8). There was an SBP reduction of 1.9, 6.1, and 15.5 mmHg among patients who did not receive any deliveries, those who received 1 delivery, and those who received 2 or more medication deliveries, respectively. Adjusted mixed-effect model estimates revealed that mean SBP reduction and self-reported medication adherence were improved among individuals who successfully received medication deliveries, compared to those who did not. A community medication delivery program in western Kenya was shown to be implementable and enhanced medication possession, reduced SBP, and significantly improved self-reported adherence. This is a promising strategy to improve health outcomes for patients with uncontrolled hypertension that warrants further investigation.

## Introduction

Cardiovascular disease (CVD) contributes significantly to the global burden of premature mortality, reduced quality of life, and increasing healthcare costs, with elevated systolic blood pressure (SBP) as the leading attributable modifiable risk factor [1, 2]. Despite many evidence-based interventions to manage hypertension, substantial implementation gaps of these interventions persist, disproportionately so in LMICs [3].

Adherence to hypertensive medication reduces blood pressure (BP) [4], congestive heart failure [5], CVD risk [6], CVD events [7, 8], and mortality [7–10]. However, fifty to sixty percent of hypertensive patients in low-resource settings worldwide report non-adherence to their medication for hypertension [11, 12]. In these settings, specific barriers to patient-level adherence are attributed to health system challenges such as unreliable medication availability [13], low affordability [13], inadequate insurance coverage [14], transportation cost [15, 16], and distance to health facilities [17, 18].

A well-functioning supply chain system for antihypertensive medications is crucial in ensuring optimal medication possession and adherence for hypertensive patients [19]. Our team at the Academic Model Providing Access to Healthcare (AMPATH) partnership has implemented numerous community-centric and health system-responsive strategies over the past decade to establish a reliable, consistent, and accountable supply chain system for essential cardiovascular disease medications [20–23]. We have improved the availability of essential CVD medications from <30% to >90% across our catchment area in western Kenya [20]. However, despite these successes in system-level supply, gaps remain with respect to patient-level possession of antihypertensive medications, leading to suboptimal adherence and worse blood pressure control [21].

Despite several evidence-based interventions to improve medication adherence for CVD, studies examining the feasibility, acceptability, and implementation of these evidence-based strategies, especially within the local context in low- and middle-income countries, are still lacking [11, 24]. Whereas strategies to enhance HIV medication adherence, including task-shifting to non-clinicians, community-based adherence support, and community-based medication delivery through differentiated care models, have been tested in randomized controlled trials, these strategies have yet to be widely implemented and evaluated in CVD management [25]. Therefore, recognizing these similar patient-level challenges to medication access, in 2019, our chronic disease management program in rural western Kenya piloted a community medication delivery program, coupled with blood pressure monitoring and self-reported adherence evaluation, for hypertensive patients. The goal of the program aimed to remove some of the aforementioned structural barriers to medication possession and ensuring consistent access to antihypertensive medications. We leveraged lessons learned through evidence-based HIV community-centered care strategies as described above to design our community-based medication delivery program for hypertension medications. Here, we describe the implementation of this pilot program and summarize the impact of this program on patient-level medication possession, self-reported medication adherence, and SBP reduction.

## Materials and methods

### Program setting

AMPATH is an academic partnership between Moi University College of Health Sciences, Moi Teaching and Referral Hospital (MTRH), and a consortium of North American universities [26]. In 2011, AMPATH established a Chronic Disease Management (CDM) program in collaboration with the Kenya Ministry of Health (MOH) to provide care for non-communicable diseases in western Kenya, enrolling over 50,000 patients since that time [27]. The CDM program provides multi-component clinical care for patients primarily at health facilities [20, 28–31], and also at the community level through a group medical visit model entitled Bridging Income Generation with grouP Integrated Care (BIGPIC) [32, 33]. BIGPIC Family extends the BIGPIC model of care to encompass comprehensive primary care to be provided by family medicine-trained clinicians in the community. Patients are identified through community-based screening and then form community-based microfinance groups of 10–30 individuals. Each group meets once biweekly to conduct microfinance group activities at a convenient location in the community. In addition, the BIGPIC Family model delivers a full complement of portable clinical services for chronic diseases such as diabetes and hypertension, in addition to services for acute ailments like malaria and pneumonia. The BIGPIC Family model is also supported by the Revolving Fund Pharmacy (RFP) scheme to provide a reliable supply of antihypertensive and antidiabetic medications to patients receiving care from the program [20, 22]. Through this scheme, all BIGPIC Family patients who are verified as active National Hospital Insurance Fund (NHIF) beneficiaries may receive medications through a memorandum of understanding between the program and our local tertiary hospital partner, without out-of-pocket payments. The BIGPIC Family model has been ongoing within a catchment area of 15,000 people in a rural setting of Bungoma County, Kenya, since 2016.

### Design of the pilot community-based medication delivery program

**Fig 1:**
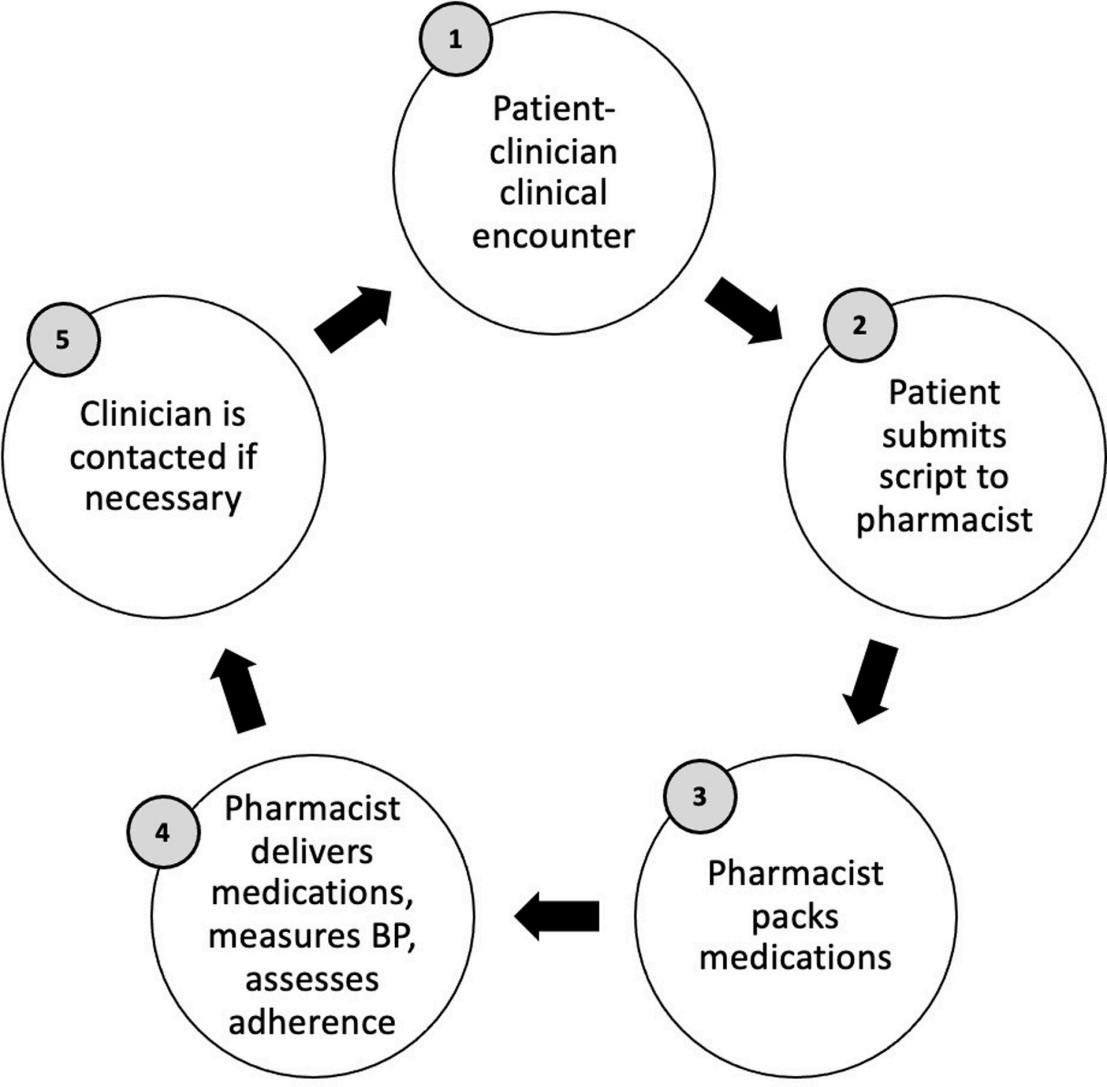
Summarized depiction of the medication delivery process.

In early 2019, recognizing the challenges faced by patients to acquire medication refills at the health facilities due to transportation costs, lost time from work, and inability to consistently pay for a full prescription out-of-pocket at the time of their clinical visits, the BIGPIC Family program developed a pilot program of community-based medication delivery to increase access to CDM medications in between clinic visits (Fig 1).

All patients 18 years and above with a diagnosis of hypertension who were receiving care from the program were invited to participate in this pilot medication delivery initiative. Patients with identified hypertensive urgencies or emergencies, as clinically evaluated by the clinician, were excluded from the pilot initiative due to their needs for more immediate and frequent interaction with a clinician. Briefly, the community-based medication delivery program was designed so that a pharmacist directly delivered medications to patients at a conveniently located medication drop-off point in the community. First, during routine clinical visits, the BIGPIC Family pharmacist, working in consultation with the program clinician, sensitized all eligible patients to the medication delivery service offered by the program. Eligible patients could initiate requests for medication deliveries at the point of their clinical visits or later via a telephone call to the pharmacist. Having a mobile phone was not a requirement for medication delivery. If a patient did not have a primary mobile phone, a secondary mobile phone number (i.e., a community health worker, a family member, a peer), as provided by the patient was recorded for contacting purposes. The names and quantities of medication supply were verbally communicated, confirmed, and verified by the pharmacist. Upon these requests, the pharmacist then pre-packed medications in opaque brown bags to conceal the specific types of medicines inside the bags and ensure patient confidentiality. The date, time, and location of delivery were also agreed upon by the patient and the pharmacist who was conducting the delivery at the time the request for medication delivery was made. The pharmacist would travel to medication drop-off points such as a patient’s individual home, a local church, a local dispensary, or any other agreed upon public locations that the patient could easily access by foot. The travel distance from the pharmacy to the patient’s drop-off point ranged between 5–35 km, and the motorcycle travel time ranged between 15–50 min. While baseline BP was measured during routine clinical appointments with the clinician, other BP checks as well as self-reported medication adherence were assessed by the pharmacist at the point of medication delivery. If the patient was present at the time of the medication delivery, the pharmacist also took the patient’s resting blood pressure using an automatic blood pressure monitoring device (Omron upper arm blood pressure monitor, 3 series) and asked the patient about their medication adherence using a 3-item adherence questionnaire [S1 File] [34]. If the patient was not present, a designated receiver, as agreed and allowed by the patient, collected the medications for the patient on their behalf. Patients who enrolled in the program but did not receive any medication deliveries provided a reason for non-delivery, which was documented by the program. For the medications that were pre-packed but could not be delivered to patients, the medications were returned to the pharmacy and were accounted for according to the pharmacy standard operating procedures. Patients who had active national health insurance received the medications at no cost, and patients without health insurance paid for the medications via cash payment or mobile money deposit directly into the pharmacy account through MPESA®.

### Statistical analysis

Routine clinical data (i.e., baseline BP, follow-up BP) were collected on electronic clinical records and paper-based pharmacy records, every time a patient had an encounter with a clinician or a pharmacist as part of clinical care provision (i.e., a clinical visit, a medication delivery.) The anonymized data set used for the analysis of this study were extracted from these sources [S1 Dataset]. We calculated the number of days during which the patients had possession of medications. Self-reported adherence was calculated by computing the mean score of the 3-item questionnaire, with higher scores representing more nonadherence. We summarized the number of days of medication possession, change in medication adherence, and change in SBP over the 6-month pilot period by mean and standard deviation. In addition, we compared differences in self-reported medication adherence and BP reduction between patients who received medication deliveries versus those who did not. Finally, we compared differences in self-reported medication adherence and BP reduction by number of medication deliveries received using a linear mixed effect model, controlling for age, gender, health insurance status, and baseline values of the outcome measure. Statistical analysis was conducted using R software (version 4.0.3; R Foundation for Statistical Computing, Vienna, Austria).

### Ethical approval

This study was approved by the Moi Teaching and Referral Hospital/Moi University Institutional Research and Ethics Committee, Eldoret, Kenya (Reference IREC/2018/76, Approval Number 0003022). The retrospective record review contains data from the medication delivery attempts from our BIGPIC Family care program. Patient consent was waived by the committee due to the retrospective and anonymized nature of all data used for analysis in the study.

## Results

### Baseline and follow-up characteristics

A total of 128 patients with a clinical indication of hypertension (80.5% women) participated in our community-based medication delivery program. Average age of participants was 59 years. Only 14.1% of the patients were actively enrolled in the Kenya National Health Insurance Fund (NHIF). At baseline, defined as the BIGPIC Family clinical encounter at which medication deliveries were requested, the average SBP 155.7 mmHg, DBP was 94.8 mmHg mmHg, with an average adherence score of 2.7 on a scale of 1–5 (1 = perfectly adherent and 5 = non-adherent) (Table 1).

**Table 1:** Baseline characteristics of all participants (N = 128), stratified by number of delivery attempts.

Characteristics	All participants	0 successful delivery	1 successful delivery	≥ 2 successful deliveries
	N = 128	n = 60 (46.9%)	n = 43 (33.6%)	n = 25 (19.5%)
Sex				
female, n (%)	103 (80.5)	54 (90.0)	32 (74.4)	17 (68.0)
male, n (%)	25 (19.5)	10 (10.0)	11 (25.6)	8 (32.0)
Age (years), mean (SD)	59.5 (13.2)	59.9 (14.4)	60.3 (12.3)	57.0 (12.0)
missing, n (%)	5 (3.9)	4 (6.5)		1 (1.6)
Insured by NHIF, n (%)	18 (14.1)	4 (6.7)	8 (18.6)	6 (24.0)
Baseline SBP (mmHg), mean (SD)	155.7 (20.3)	158.8 (19.8)	151.0 (19.8)	155.7 (21.6)
missing, n (%)	3 (2.3)		3 (7.0)	
Baseline DBP (mmHg), mean (SD)	94.8 (13.8)	97.2 (13.0)	90.3 (14.0)	96.2 (14.4)
missing, n (%)	3 (2.3)		3 (7.0)	
Self-reported adherence score*, mean (SD)	2.7 (1.7)	3.0 (1.9)	2.1 (1.5)	2.8 (1.6)
missing, n (%)	29 (22.7)	9 (15.0)	16 (37.2)	4 (16.0)

*(1 = perfect adherence, 5 = non-adherence)

Of those who participated, 43 (33.6%) patients received 1 successful medication delivery and 25 (19.5%) received 2 or more successful deliveries. We were not able to make medication deliveries to 60 (46.9%) of the patients. Of the 70 delivery attempts made to these patients, common reasons for unsuccessful deliveries included: patient was not reachable via telephone (52.9%), patient did not have enough cash to pay for drugs (24.3%), patient reported purchasing medicines elsewhere (12.9%), patient reported having enough medicines (5.7%), and other reasons (4.3%) [S2 Dataset. Reasons for unsuccessful delivery attempts].

Follow-up systolic blood pressures, diastolic blood pressures, and self-reported adherence scores, stratified by number of delivery attempts are also summarized in Table 2.

**Table 2:** Follow-up blood pressure measurements and self-reported adherence scores, stratified by number of delivery attempts.

Characteristics	All participants	0 successful delivery	1 successful delivery	≥ 2 successful deliveries
	N = 128	n = 60	n = 43	n = 25
**Follow-up outcome measurements**				
Follow up SBP (mmHg), mean (SD)	145.3 (21.1)	158.2 (18.6)	145.1 (19.1)	140.4 (23.8)
missing, n (%)	53 (41.4)	50 (83.3)	2 (4.7)	1 (4.0)
DBP	89.6 (12.4)	90.4 (9.2)	88.5 (13.5)	91.1 (11.7)
missing, n (%)	53 (41.4)	50 (83.3)	2 (4.7)	1 (4.0)
Self-reported adherence score	2.3 (1.8)	3.3 (1.7)	2.2 (1.8)	1.8 (1.6)
missing, n (%)	45 (35.2)	44 (73.3)	1 (2.3)	0 (0.0)
**Outcome changes** (Δ) **over time (follow-up–baseline)**				
Δ SBP	-8.6	-1.9	-6.1	-15.5
missing, n (%)	56 (43.8)	50 (83.3)	5 (11.6)	1 (4.0)
Δ DBP	-2.9	-5.0	-1.2	-4.6
missing, n (%)	56 (43.8)	50 (83.3)	5 (11.6)	1 (4.0)
Δ Self-reported adherence score	-0.3	+0.5	-0.3	-0.8
missing, n (%)	66 (48.4)	46 (76.7)	16 (37.2)	4 (16)

### Duration of medication possession

Antihypertensive medication possession (i.e. patient had at least one antihypertensive medication in their hands to facilitate taking the medication as prescribed) was 29 days for patients who received 0 successful deliveries, 70 days for patients receiving 1 successful delivery, and 151 days for 2 or more successful deliveries.

### Change in self-reported medication adherence

**Fig 2:**
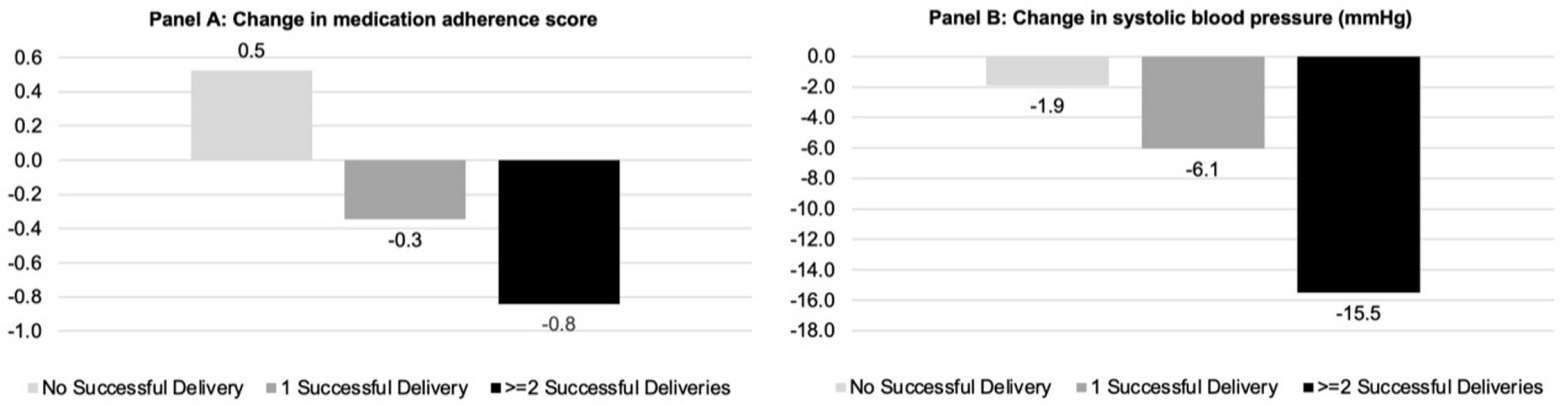
Change in self-reported medication adherence score and systolic blood pressure measurement.

Using a 3-item questionnaire (Voils DOSE-Nonadherence measurement), we observed a +0.5-point increase in those who did not receive any medication deliveries, indicating worse medication adherence. In contrast, there was a -0.3-point reduction and -0.8-point reduction, indicating improved adherence, in those who received 1 successful delivery and 2 or more successful deliveries, respectively (Fig 2, Panel A). Adjusted mixed-effect model estimates revealed that there was a statistically significant improvement in self-reported medication adherence among individuals who successfully received greater number of medication deliveries. Compared to those who had no successful delivery, those who received 1 successful delivery had a -1.2 (95% confidence interval [CI] -2.3 to -0.2, p = 0.03) lower score (indicating better adherence), and those who received 2 successful deliveries had -1.3 (95% CI -2.3 to -0.3, p = 0.01) lower score (Table 3).

**Table 3:** Mixed-effect model estimates.

	Self-reported adherence score			Systolic blood pressure			Diastolic blood pressure		
				(mmHg)			(mmHg)		
	Estimate	95% CI	*p*-value	Estimate	95% CI	*p*-value	Estimate	95% CI	*p-value*
**1 successful delivery vs.**	-1.2	(-2.3, -0.2)	0.03*	-8.1	(-20.8, 4.6)	0.21	2.43	(-5.1, 9.95)	0.53
**No successful delivery**									
**≥2 successful deliveries vs.**	-1.3	(-2.3, -0.3)	0.01*	-10.8	(-23.6, 2.0)	0.10	2.75	(-4.8, 10.3)	0.48
**No successful delivery**									

* Indicates statistical significance

### Change in systolic blood pressure

Patients who received 2 or more medication deliveries had the largest SBP reduction of -15.5 mmHg compared to baseline, while those who received 1 medication delivery had an SBP reduction of -6.1 mmHg, and those who received no medication delivery had a reduction of -1.9 mmHg (Fig 2, Panel B). Adjusted mixed-effect model estimates revealed that there was a trend toward greater SBP reduction among individuals who successfully received greater number of medication deliveries. Compared to those who had no successful delivery, those who received 1 successful delivery demonstrated SBP reduction of -8.1 mmHg (CI -20.8 to 4.6, p = 0.21), and those who received 2 successful deliveries had SBP reduction of -10.8 mmHg (CI -23.6 to 2.0, p = 0.10) (Table 2).

## Discussion

The retrospective assessment of our pilot community-based program demonstrated that successful medication delivery to patients increased patient-level medication possession, and improved medication adherence. While we observed a trend towards better blood pressure reduction among patients who received greater number of deliveries, this was not statistically significant. The findings generated by this study contribute to the evidence on the implementation of community-based models of medication deliveries for hypertension and other cardiovascular diseases, especially in resource-limited settings [25].

Inadequate medication possession, acquisition, and refills have been linked to suboptimal medication adherence [35–37]. Our community-based medication delivery program for antihypertensive medications was established to enable patients to consistently acquire needed antihypertensive medications, at the time when they could access and afford these medicines. We leveraged and adapted the designs from other community-based medication delivery programs that have primarily been studied in patients living with HIV/AIDS in Africa, such as community antiretroviral therapy (ART) distribution [38], medication adherence clubs [39, 40], and community ART groups [41]. In the HIV patient population, these initiatives are part of the differentiated care model to decouple the process between an ART medication refill encounter and a clinical encounter/consultation for patients who are otherwise stable on their current ART regimen [25]. By taking the same approach, our medication delivery program for antihypertensive medications allowed for patients to acquire medications without having to use their limited resources to travel to a health facility to purchase medications, pay for transportation to get to a pharmacy, and/or wait for long hours to see a clinician and get the prescribed medications, all of which have been described as barriers to medication access [15–18, 42] In addition, by leveraging the Revolving Fund Pharmacy infrastructure which consistently availed cardiovascular disease medications, we ensured that medications were always reliably available to supply to patients and their needs [20, 22]. As a result, we have been able to demonstrate that medication possession was higher among patients who received greater number of medication deliveries, thus improving the patients’ ability to have medications in their hands and to take these medicines as prescribed.

Patients who received successful medication deliveries reported significantly improved medication adherence, as compared to those who received no medication deliveries. These results could potentially be explained by three mechanisms. First, by simply removing structural barriers to access through medication deliveries to patient’s preferred locations in their communities, it was easier and more convenient for patients to acquire medications, thus making medication adherence more achievable [21, 23]. In a recent patient preference study conducted by South Africa by Adams et al., 80.2% of study participants were interested in a chronic disease medicine delivery service, including essential CVD medications, that would deliver medicines to their homes, and most of the patients (77.6%) were willing to pay for the service [43]. Second, the indirect cost savings from transportation costs and time savings to reach a health facility or pharmacy may have made it more affordable for patients to acquire the drugs, despite their constraint resources. Third, the pharmacist who acted as the medication delivery agent, transporting medications from the health facility pharmacy to the patient home, also provided hypertension care, adherence counseling, and social support to these patients as part of an interdisciplinary care team within the BIGPIC Family model. As such, the pharmacist is a trusted healthcare provided who is well-known and respected by the patients. Optimizing patient-provider relationships to increase trust between patients and clinicians have long been supported as an evidence-based intervention to improve medication adherence [44].

With respect to SBP lowering in relation to medication delivery, results from our model estimates, while encouraging, did not achieve statistical significance. We hypothesize that this was because of our relatively small sample size and short study duration. Nevertheless, blood pressure lowering has been shown to be associated with long-term cardiovascular benefits [45]. As there is currently limited evidence regarding the efficacy of medication delivery programs and their impact on biological outcomes for patients living with non-communicable disease such as hypertension, our study adds to the current evidence base, and supports the implementation of a more rigorous, adequately powered, definitive trial [25].

Many of our patients who initially expressed interest in participating in our pilot program did not receive any successful deliveries. These patients reported financial and structural difficulties preventing them from successfully receiving medication deliveries. Future iterations of this intervention should consider addressing these challenges to ensure equitable reach to all patients, particularly those facing significant lack of resources. For example, health financing policies should emphasize the inclusion of essential cardiovascular medicines in their outpatient benefit package through social or national health insurance to ensure optimal medication affordability for patients [23]. Nevertheless, lessons learned with respect to reasons for unsuccessful deliveries from this pilot program are important for future adaptations and implementation efforts. We anticipate that other programs trying to replicate our intervention can utilize our pilot description and results to better understand the context in which this study was conducted and guide their own implementation.

## Limitations

This study was not without its limitations. First, we were limited to medication delivery data from our program. We were unable to track if patients procured medications elsewhere (i.e. a private pharmacy, a local dispensary). Thus, we could not calculate true medication possession ratios, defined as the proportion of a time period when medication supply is available. Second, visual inspection of medications or pill counting could have helped to supplement the self-reported adherence questionnaires. Third, this pilot initiative was conducted on a small cohort of patients who self-selected to participate in the program with a relatively short duration of follow-up. A more rigorous randomized controlled trial is needed to fully reveal biological as well as process outcomes. Finally, we acknowledge that not every clinical program will have the human resource capacity to have one full-time pharmacist be responsible for the entire process ranging from medication packaging to ultimate delivery. However, given its encouraging feasibility and outcomes, it is possible to engage other trusted health providers in the community such as peers and community health workers to become medication delivery agents and support the expansion of this pilot program.

## Conclusions

A community medication delivery program in western Kenya was shown to be implementable and enhanced medication possession, reduced SBP, and significantly improved self-reported adherence. This is a promising strategy to improve health outcomes for patients with uncontrolled hypertension that warrants further investigation.

## Supporting information

S1 File Voils DOSE-Nonadherence measurement. (DOCX)

S1 Dataset De-identified anonymous minimal data set. (XLSX)

S2 Dataset Reasons for unsuccessful delivery attempts. (XLSX)
