# Supplementary material for: Community-based medication delivery program for antihypertensive medications improves adherence and reduces blood pressure
Source: PLoS One. 2022 Sep 9;17(9):e0273655. doi: 10.1371/journal.pone.0273655 (PMC9462824; doi:10.1371/journal.pone.0273655)
Supplement: S1 File — (DOCX) [file pone.0273655.s001.docx]

**S1 Questionnaire – Voils DOSE-Nonadherence Measurement**

Over the past 7 days…

1. I took all doses of my medication.
2. I missed or skipped at least one dose of my medication.
3. I was not able to take all of my medication.
